# Identification of two *Wolbachia* genes with cell proliferation-inhibitory activity in *Ostrinia* cultured cells

**DOI:** 10.1128/mbio.00074-26

**Published:** 2026-03-30

**Authors:** Susumu Katsuma, Noriko Matsuda-Imai, Tomohiro Muro, Mayuko Yoda, Shinpei Kawaoka, Takashi Kiuchi

**Affiliations:** 1 Department of Agricultural and Environmental Biology, Graduate School of Agricultural and Life Sciences, The University of Tokyo 13143, Bunkyo-ku, Tokyo, Japan; 2 Department of Insect Symbiosis, Max Planck Institute for Chemical Ecology 28298, Jena, Germany; 3 Department of Integrative Bioanalytics, Institute of Development, Aging and Cancer (IDAC), Tohoku University 13101, Sendai, Japan; Sangyo Gijutsu Sogo Kenkyujo Hokkaido Center, Sapporo, Hokkaido, Japan

**Keywords:** *Wolbachia*, lepidopteran insects, transinfection, functional gene screening, *TomO* homolog

## Abstract

*Wolbachia* species are endosymbiotic bacteria that regulate the sexual development and reproduction of their insect hosts. Despite their biological importance, functional analyses of *Wolbachia* genes remain limited, largely due to the absence of a robust genetic manipulation system for these intracellular bacteria. Here, we examined the impact of *w*Fur, a *Wolbachia* strain originally isolated from *Ostrinia furnacalis*, on host cell proliferation. Inoculation of *w*Fur onto *Wolbachia*-free *O. furnacalis* cultured cells resulted in pronounced inhibition of cell proliferation, accompanied by cytotoxic effects. To identify the genetic determinants of this phenotype, we conducted functional screening of *w*Fur-derived genes in *O. furnacalis* cultured cells. This approach identified two candidate genes whose overexpression markedly suppressed cell proliferation. One gene, designated *w52*, encodes a protein with strong sequence and structural homology to TomO (toxic manipulator of oogenesis), previously reported to inhibit *Drosophila* S2 cell proliferation. The second gene, designated *w75*, encodes a putative membrane-associated protein widely conserved among *Wolbachia* genomes. Moreover, overexpression of *w52* or *w75* also impaired cell proliferation in *Bombyx mori* cultured cells, whereas no effect was observed in *Spodoptera frugiperda* Sf-9 cells. These results suggest that the cytostatic activity of these *Wolbachia* genes is likely to be insect species- and/or cell type-specific.

**IMPORTANCE:**

*Wolbachia* are maternally inherited intracellular bacteria notable for their exceptionally broad distribution among insect species. Despite their importance, particularly as reproductive manipulators, only a limited number of studies have addressed the functions of *Wolbachia* genes, largely due to the absence of well-established transinfection systems and reliable genetic engineering tools for these intracellular bacteria. In this study, we developed a transinfection system using *Ostrinia furnacalis* cultured cells and its male-killing strain *w*Fur. Moreover, we identified two *w*Fur genes, whose expression inhibited cell proliferation and exhibited cytotoxicity, through an in-house functional screening system of *w*Fur genes. This strategy can be readily extended to investigations of other *Wolbachia* species, thereby contributing to a more comprehensive understanding of *Wolbachia* gene functions.

## INTRODUCTION

*Wolbachia* rank among the most widespread intracellular bacteria known to manipulate host insect reproduction. They enhance the transmission of infected females through multiple reproductive modifications, including parthenogenesis induction, feminization, cytoplasmic incompatibility (CI), and male killing (MK). Each of these strategies is regarded as evolutionarily advantageous for *Wolbachia*, promoting their long-term persistence and dissemination within host populations (1, 2). To date, several effector genes involved in reproductive manipulation have been functionally characterized, mostly through transgenic or transient expression in host insects (3–5) and overexpression in cultured cells (5–7).

The CI-inducing genes *cifA* and *cifB* (also referred to as *cidA* and *cidB* in the *w*Pip strain) were identified by comparative genomics of several *Wolbachia* strains. Transgenic studies using *Drosophila melanogaster* and *Anopheles gambiae* (3, 8), along with biochemical and structural analyses of the CifA–CifB complex (9–11), have elucidated the molecular mechanism by which CifB-induced toxicity is rescued by CifA expression in females. Regarding MK effectors, two genes have been identified: *wmk* (*WO-mediated killing*) and *Oscar* (*Osugoroshi protein containing CifB C-terminus-like domain and many ankyrin repeats*) (4, 5, 12, 13). Transgenic expression of *wmk* in *D. melanogaster* induces embryonic lethality, resulting in a significant female-biased sex ratio (average male:female = 0.65:1) (4). On the other hand, Oscar was identified through biochemical approaches employing cultured cells derived from the Asian corn borer *Ostrinia furnacalis* infected with the male-killing *Wolbachia* strain *w*Fur (5). Oscar binds to the host masculinizing factor OfMasc and promotes its degradation via the host proteasome pathway. Transient expression of *Oscar* mRNA in embryos phenocopied MK in *O. furnacalis* (5, 13). Beyond these effectors, a putative gene, *piff* (*parthenogenesis-induction feminization factor*), linked to *Wolbachia*-induced parthenogenesis has recently been identified in the *Wolbachia* genome of the haplodiploid parasitoid wasp *Encarsia formosa*. This gene encodes a homolog of the host *transformer* (*tra*) gene, and its gene product interacts with host Transformer-2 (TRA2), presumably inducing feminization (7, 14).

While significant progress has been made in elucidating the molecular basis of *Wolbachia*-mediated sexual and reproductive manipulations, the functional characterization of other *Wolbachia* genes remains extremely limited (15–18), primarily due to the lack of a reliable genetic manipulation system for *Wolbachia*. Using surrogate organisms, the functions of several *Wolbachia* genes have been explored. *TomO* (*toxic manipulator of oogenesis*) was identified through a genomic screen for *Wolbachia w*Mel genes that exhibit proliferation-inhibitory effects on *D. melanogaster* S2 cells (15). Further analysis using transgenic *D. melanogaster* revealed that TomO homologs of *w*Mel and *w*Pip enhance the maintenance of germ stem cells by elevating Nanos expression (15, 19). Recently, two *Wolbachia* genes *Wbm0076* and *wBm0152*, identified from *Wolbachia* of human pathogenic filarial nematodes, were characterized using the yeast *Saccharomyces cerevisiae*. Genetic analyses revealed that Wbm0076 likely plays a role in the active cell-to-cell movement of *Wolbachia* (16). Additionally, the same research group reported that exogenous expression of wBm0152, a *Wolbachia* outer membrane lipoprotein, strongly disrupted endosomal maturation, leading to defects in ubiquitylated protein turnover in *S. cerevisiae* (17). Martin et al. identified a *Wolbachia* effector, WalE1, that increases *Wolbachia* abundance in the next generation in transgenic *D. melanogaster* (18). The WalE1 protein contains an N-terminal alpha-synuslein domain, which interacts with the host protein Past1. Further experiments using *S. cerevisiae* and *D. melanogaster* expressing WalE1 revealed that this protein alters host endocytosis (18).

In our ongoing investigation of the MK mechanism in *O. furnacalis* and its associated *Wolbachia* strain *w*Fur (5), we established a transinfection system and observed that inoculation of *O. furnacalis* cultured cells with *w*Fur-containing solution resulted in pronounced inhibition of cell proliferation. To identify the gene(s) responsible for this phenotype, we performed functional screening using *w*Fur gene constructs in *O. furnacalis* cultured cells. This approach led to the identification of two candidate genes exhibiting strong inhibitory activity in cell proliferation.

## RESULTS

### *Wolbachia* infection inhibits the proliferation of *O. furnacalis* cultured cells

**Fig 1:**
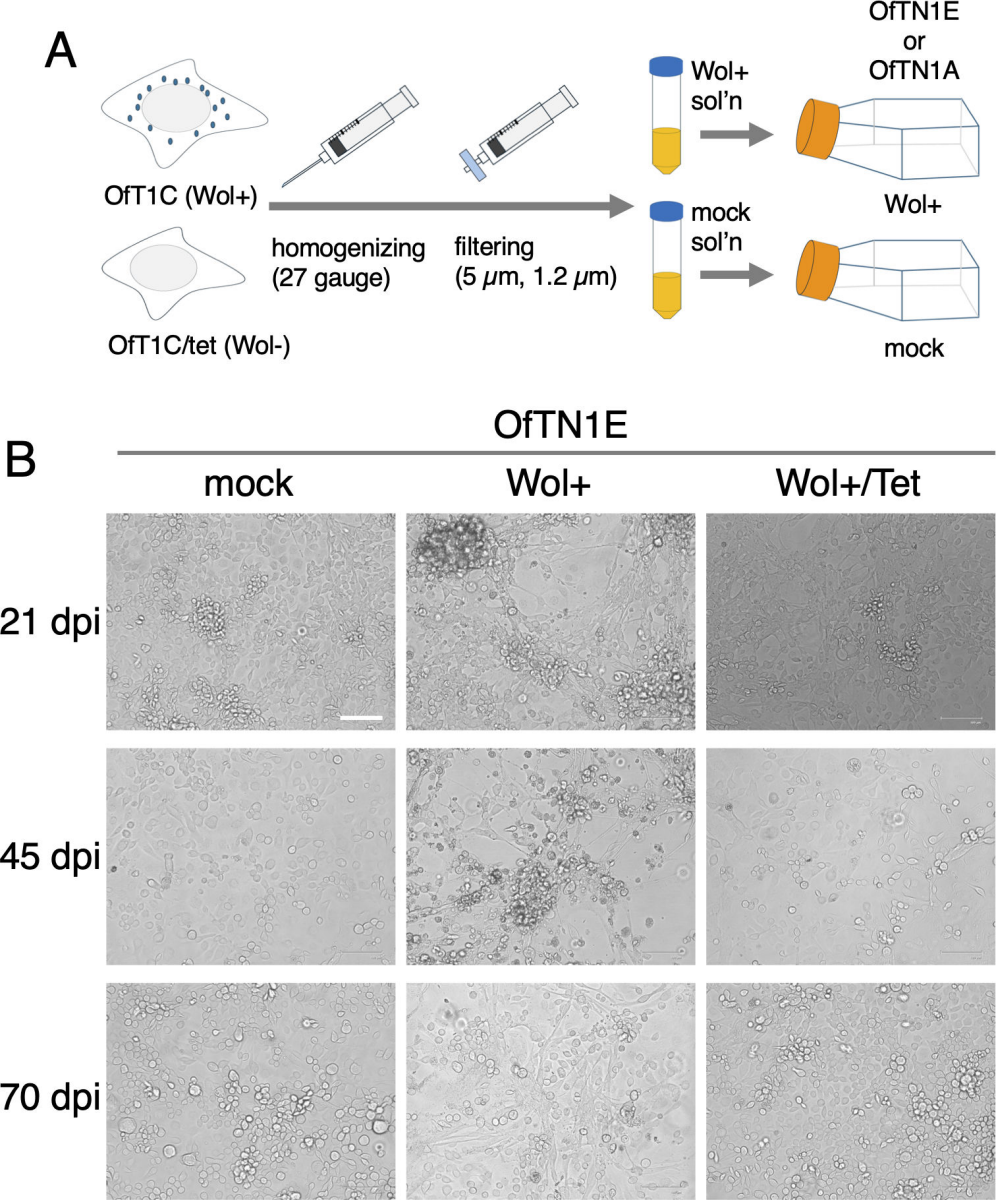
*w*Fur infection inhibits the growth of *O. furnacalis* OfTN1E cells. (**A**) Transinfection scheme of *w*Fur using *O. furnacalis* cultured cells. A *w*Fur-containing solution was prepared from *w*Fur-infected OfT1C cells and inoculated onto *Wolbachia*-free OfTN1E or OfTN1A cells. A *Wolbachia*-free solution prepared from *Wolbachia*-free OfT1C/tet cells was used as a negative control. (**B**) Growth of OfTN1E cells following *w*Fur infection. OfTN1E cells were inoculated with *w*Fur solution or mock solution. The medium was replaced with fresh medium with or without tetracycline, and cells were photographed at 21, 45, and 70 dpi. Bar, 100 µm.

We sought to develop a method for *Wolbachia* transinfection in lepidopteran cells using *w*Fur. As illustrated in Fig. 1A, a *w*Fur-containing solution was prepared from OfT1C cells, established from *w*Fur-infected *O. furnacalis* embryos (5), and subsequently inoculated onto OfTN1E cells, a newly established cell line originating from *Wolbachia*-free *O. furnacalis* embryos. As a negative control, we employed a solution derived from *Wolbachia*-free OfT1C/tet cells (5). Following *w*Fur inoculation, OfTN1E cells exhibited pronounced inhibition of cell proliferation and morphological abnormalities, including aberrant elongation and aggregate formation, which were particularly evident at 45 days post-infection (dpi) (Fig. 1B). These phenotypic abnormalities were partially alleviated by 70 dpi. In contrast, treatment with the *Wolbachia*-free control solution had no effect on cell morphology or proliferation (Fig. 1B). Furthermore, administration of tetracycline 1 day after *w*Fur inoculation completely abolished the phenotypic defects (Fig. 1B), confirming that these effects were specifically attributable to *w*Fur infection.

**Fig 2:**
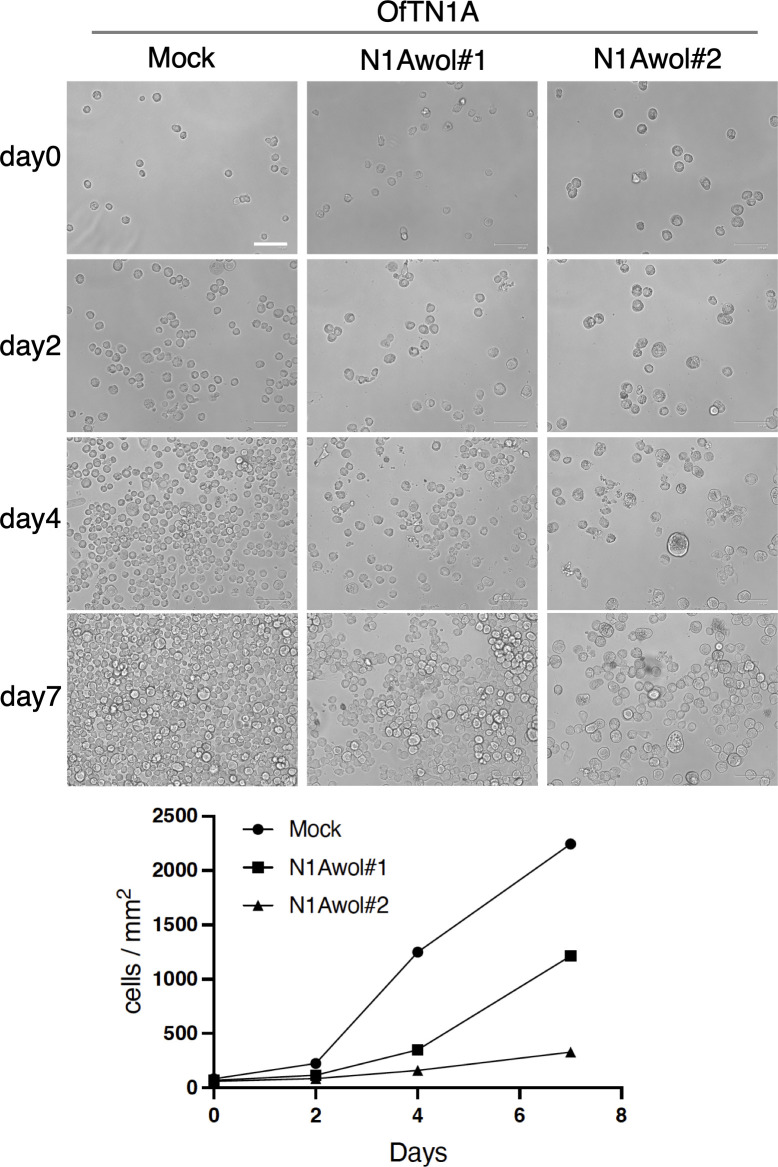
Persistent *w*Fur infection inhibits the growth of *O. furnacalis* OfTN1A cells. Equal numbers (5.0 × 10^4^ cells) of mock-infected, OfTN1Awol#1, and OfTN1Awol#2 cells were seeded in 35-mm dishes and photographed on days 0, 2, 4, and 7. Bar, 100 µm. The average cell numbers counted from 2 to 4 images are shown below.

**Fig 3:**
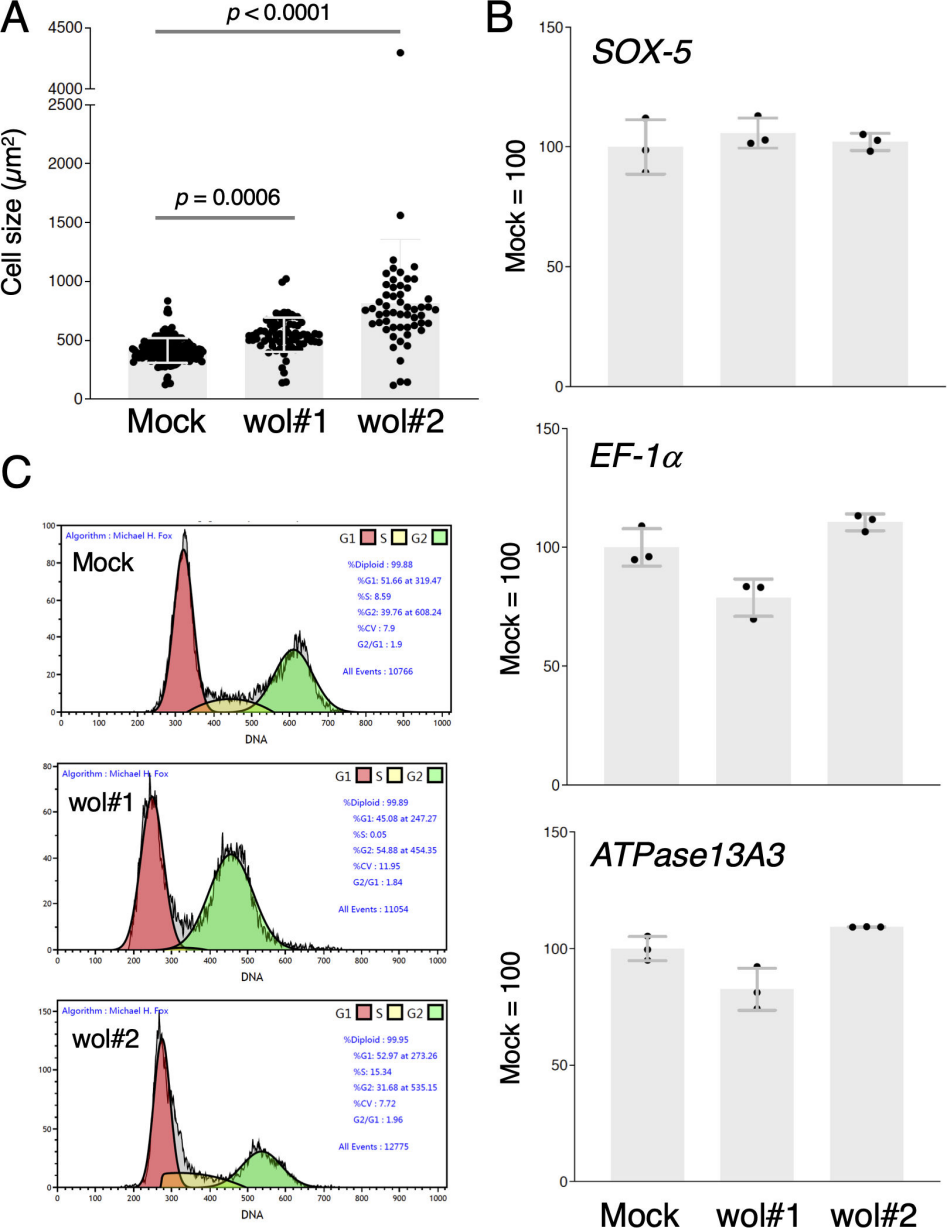
Phenotypes of persistently *w*Fur-infected *O. furnacalis* OfTN1A cells. (**A**) Measurement of cell size. The cell sizes of mock-infected, OfTN1Awol#1, and OfTN1Awol#2 cells were estimated using ImageJ. Data shown are means ± SD. Adjusted *P* values following one-way ANOVA with Tukey’s multiple comparisons tests are shown. (**B**) Comparing chromosome numbers by genomic qPCR using the primer sets targeting three autosomal genes, *SOX-5*, *EF-1a*, and *ATPase13A3*. Data shown are means ± SD of triplicate measurements. (**C**) FACS-mediated cell cycle analysis. G1, S, and G2/M cells are shown by different colors. Similar results were obtained in two independent experiments.

To further validate these observations, we examined the impact of *w*Fur infection on another *Wolbachia*-free cell line, OfTN1A. Using the same transinfection protocol (Fig. 1A), we successfully generated two persistently *w*Fur-infected cell lines, designated OfTN1Awol#1 and OfTN1Awol#2 (the latter treated with five times the amount of *Wolbachia* solution compared with OfTN1Awol#1), which have been stably maintained for more than 850 days. Both lines exhibited markedly reduced proliferation, with OfTN1Awol#2 displaying more pronounced retardation (Fig. 2). The average cell size in both infected lines was significantly larger than that of mock-inoculated, *Wolbachia*-free OfTN1A cells (Fig. 3A). Quantitative genomic PCR analysis using three autosomal genes (*SOX-5*, *EF-1α*, and *ATPase13A3*) indicated that the chromosome numbers of *Wolbachia*-inoculated cells remained unchanged (Fig. 3B). Fluorescence-activated cell sorting (FACS) analysis revealed that OfTN1Awol#1 cells exhibited distinct G2/M cell cycle arrest, whereas OfTN1Awol#2 cells did not (Fig. 3C).

**Fig 4:**
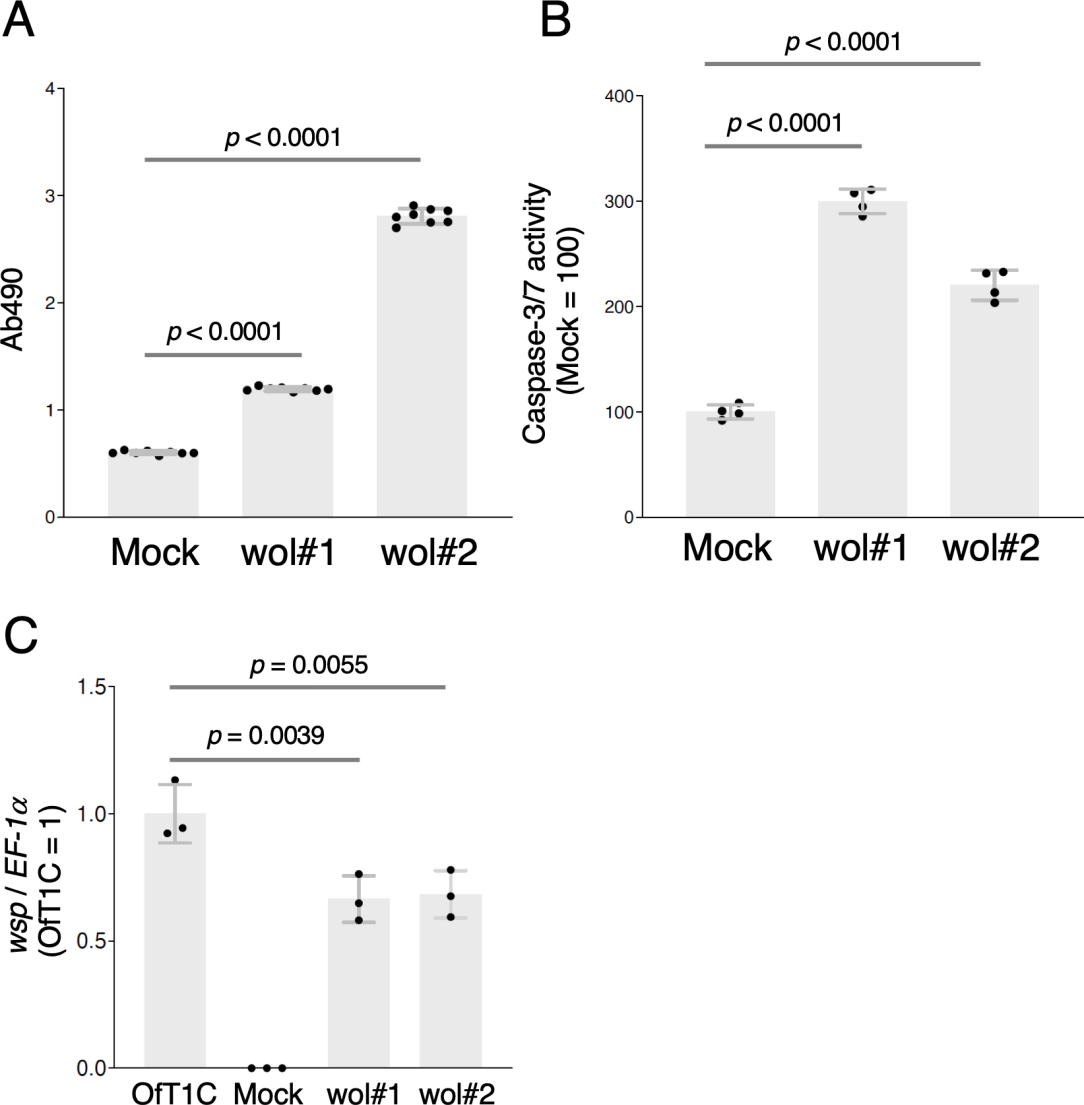
Cytotoxic effects of *w*Fur infection on *O. furnacalis* OfTN1A cells. (A–B) LDH (**A**) and caspase-3/7 (**B**) activities of OfTN1A cells infected with *w*Fur. Data shown are means ± SD of eight (LDH) and four (caspase-3/7) samples. Similar results were obtained in two independent experiments. (**C**) *Wolbachia* density. *Wolbachia* density in OfT1C, mock-infected, OfTN1Awol#1, and OfTN1Awol#2 cells was estimated by qPCR of *wsp* and normalized to *EF-1a*. Data shown are means ± SD of triplicate measurements. Similar results were obtained in two independent experiments. Adjusted *P* values following one-way ANOVA with Tukey’s multiple comparisons tests are shown.

To examine the cytotoxic effects of *Wolbachia* infection, we measured lactate dehydrogenase (LDH) and caspase-3/7 activities in OfTN1Awol#1 and OfTN1Awol#2 cells. LDH activity was significantly higher in *Wolbachia*-inoculated cells, particularly in OfTN1Awol#2, compared with mock-inoculated cells (Fig. 4A). The *Wolbachia*-inoculated cells also exhibited higher caspase-3/7 activity than mock-inoculated cells (Fig. 4B). Although OfTN1Awol#1 and OfTN1Awol#2 displayed phenotypic differences, intracellular *Wolbachia* density in both lines was comparable and slightly lower than that observed in OfT1C cells (Fig. 4C). Collectively, these results demonstrate that *w*Fur infection exerts substantial effects on the proliferation, morphology, and cytotoxicity of *O. furnacalis* cultured cells and that stable transinfection of lepidopteran cell lines with *Wolbachia* is feasible using the described methodology.

### Identification of two *w*Fur genes with inhibitory effects on cell proliferation

**Fig 5:**
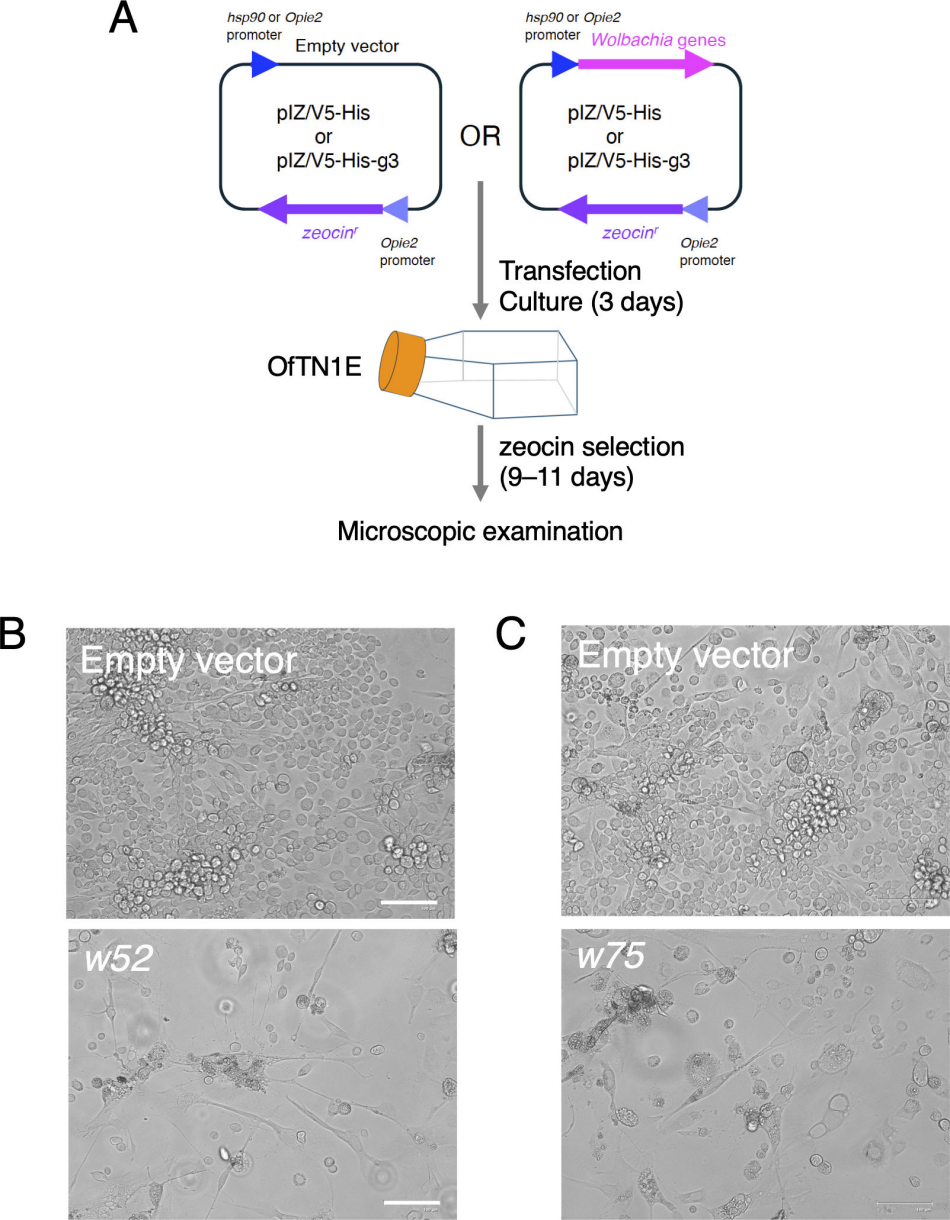
Identification of *w*Fur genes with cell proliferation-inhibitory activity in *O. furnacalis* cells. (**A**) Scheme of functional screening for *w*Fur genes with cell proliferation-inhibitory activity. OfTN1E cells were transfected with plasmids containing *w*Fur genes, selected with zeocin, and photographed at 11–13 days after transfection. (**B**) Phenotype of OfTN1E cells stably transfected with a *w52*-expressing plasmid. (**C**) Phenotype of OfTN1E cells stably transfected with a *w75*-expressing plasmid. Bar, 100 µm. See also Fig. S1.

Utilizing an expression library comprising ~300 *w*Fur-derived clones, each cloned into the insect expression vectors pIZ/V5-His-g3 or pIZ/V5-His, we performed a systematic screening to identify gene(s) that inhibit the proliferation of *O. furnacalis* cultured cells. Individual plasmids were transfected into OfTN1E cells, followed by selection of transformants with zeocin. Cell proliferation was subsequently assessed at 11–13 days post-transfection (Fig. 5A). Through this screening, we identified two clones whose overexpression resulted in pronounced inhibition of cell proliferation, abnormal elongation, and aggregate formation of transfected cells (Fig. 5B and C; Fig. S1). These phenotypic alterations closely resembled those observed in *w*Fur-inoculated OfTN1E cells (Fig. 1B), suggesting that the identified clones may harbor genes responsible for *w*Fur-induced suppression in *O. furnacalis* cell proliferation. The corresponding genes were designated *w52* and *w75* (their locus tags in the *w*Fur genome, NZ_CP096925.1, are M1L25_RS06200 and M1L25_RS02980, respectively).

**Fig 6:**
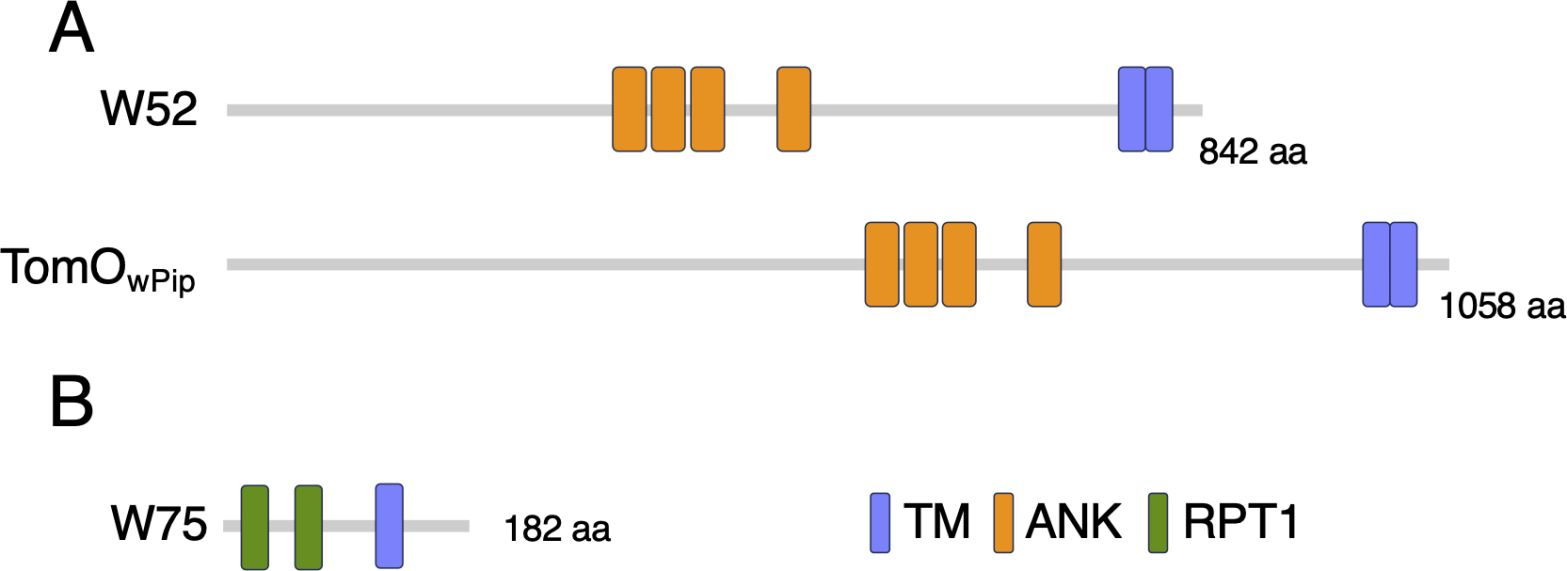
Structures of W52 and W75. (**A**) Structures of the W52 and TomO_wPip_ proteins. (**B**) Structure of the W75 protein. Domains and motifs predicted by SMART are indicated.

**Fig 7:**
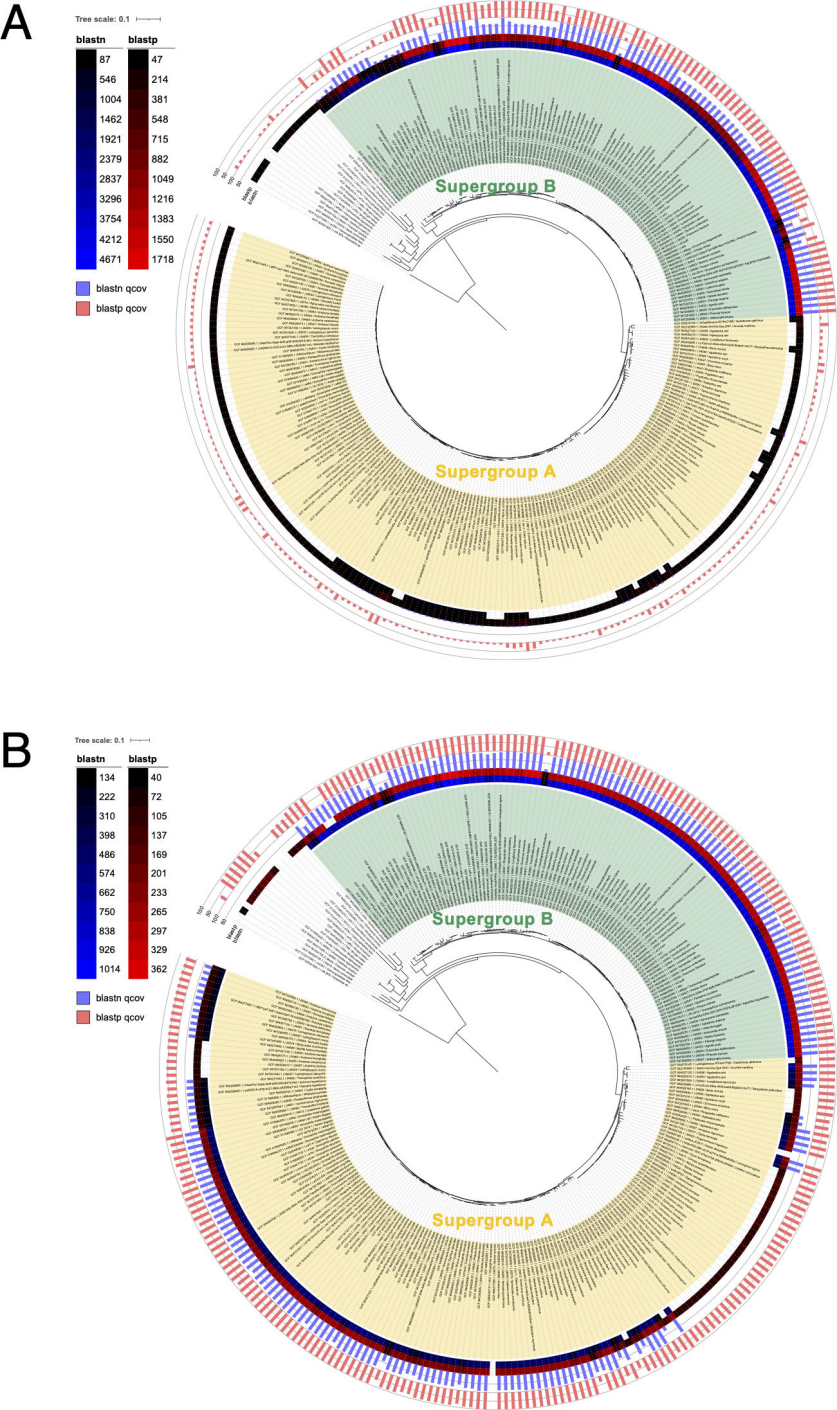
Phylogenetic analysis of W52 and W75. Phylogenetic distribution of *w52* (**A**) and *w75* (**B**) homologs across *Wolbachia* genomes. The outermost bars and heatmaps represent the query coverage and bit score, respectively, from blast analysis of *w52* and *w*75. Blank regions (white background) indicate the absence of hits. The phylogenetic tree was constructed from a concatenated alignment of 203 single-copy genes from 311 *Wolbachia* genomes and is displayed using midpoint rooting, with some lineages removed for clarity. Supergroups A and B are distinguished by different background colors. The complete trees, including all lineages, are shown in Fig. S3 (W52) and Fig. S5 *(*W75).

The cloned *w52* gene encodes a protein of 768 amino acids, corresponding to a partial sequence of an 842 aa-long *w*Fur protein (Fig. S2). This protein contains four ankyrin (ANK) repeats and two predicted transmembrane domains at the C-terminus (Fig. 6A). Sequence analysis revealed a high degree of similarity between W52 and the *w*Pip protein known as TomO (toxic manipulator of oogenesis) (15, 19) (Fig. S2; Fig. 6A). Notably, transfection of the *w*Mel *TomO* homolog has previously been shown to induce inhibition of cell proliferation in *D. melanogaster* S2 cells (15), implying conserved functional roles of *TomO* homologs across dipteran and lepidopteran species. Phylogenetic analysis revealed that the *w52* gene is well conserved within supergroup B *Wolbachia*, whereas most supergroup A *Wolbachia* lack this gene (Fig. 7A; Fig. S3).

The *w75* gene encodes a novel protein consisting of 182 amino acids (Fig. 6B). AlphaFold3-based prediction suggested that the overall W75 sequence potentially forms a very long α-helix (Fig. S4). BLAST analysis indicated that homologs of this protein are widely distributed among various *Wolbachia* strains (Fig. 7B; Fig. S5). The *w75* gene is well conserved within supergroup B *Wolbachia*, whereas certain clades within supergroup A lack this gene. Moreover, other supergroups do not possess *w75*-like genes with high sequence homology to the *w*Fur *w75* gene (Fig. 7B; Fig. S5). Similar to W52, the W75 protein also contains a predicted transmembrane domain at its C-terminal region (Fig. 6B).

**Fig 8:**
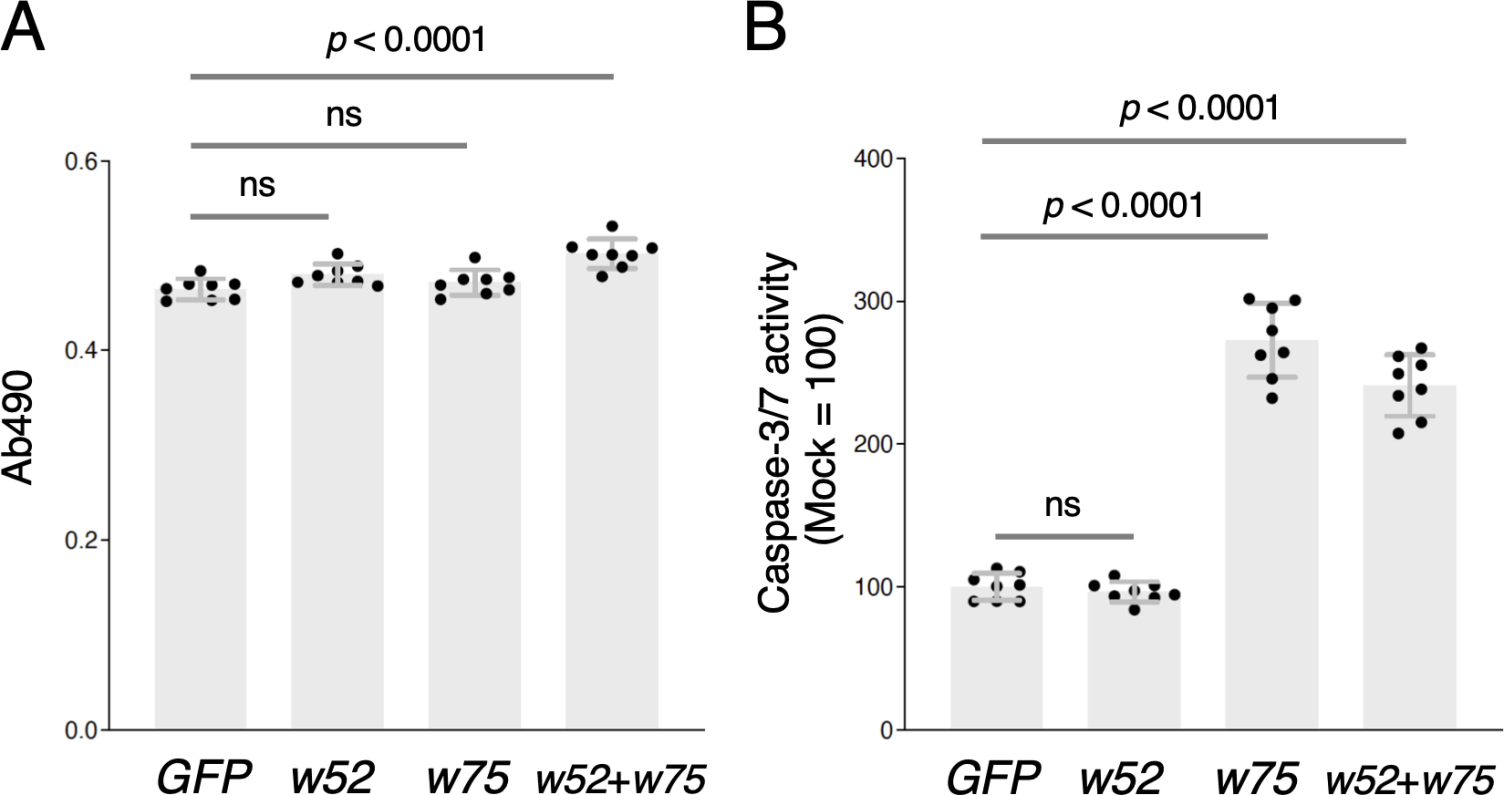
Cytotoxic effects of *w52* and *w75* expression in *O. furnacalis* cultured cells. LDH (**A**) and caspase-3/7 (**B**) activities were measured in OfTN1E cells transiently transfected with plasmids expressing *gfp*, *w52*, *w75*, and both *w52* and *w75*. Adjusted *P* values following one-way ANOVA with Tukey’s multiple comparisons tests are shown. Data shown are means ± SD of eight samples. Similar results were obtained in two independent experiments. ns, *P* > 0.05.

To investigate the cytotoxic effects of *w52* and/or *w75* expression in *O. furnacalis* cells, we measured LDH and caspase-3/7 activities in OfTN1E cells transiently expressing *gfp* (control), *w52*, and/or *w75*. LDH activity in *w52*- or *w75*-transfected cells was comparable to that in *gfp*-transfected cells, whereas transfection with both *w52* and *w75* significantly increased the activity (Fig. 8A). On the other hand, caspase-3/7 activity was significantly higher in *w75*-transfected cells compared with that in *gfp*-transfected cells (Fig. 8B). Transfection of *w52* alone did not affect the caspase-3/7 activity (Fig. 8B). These results suggest that both W52 and W75 may contribute to cytotoxic effects in *O. furnacalis* cells.

### Expression of *w52* and *w75* in *w*Fur-infected *O. furnacalis* pupae and cultured cells

**Fig 9:**
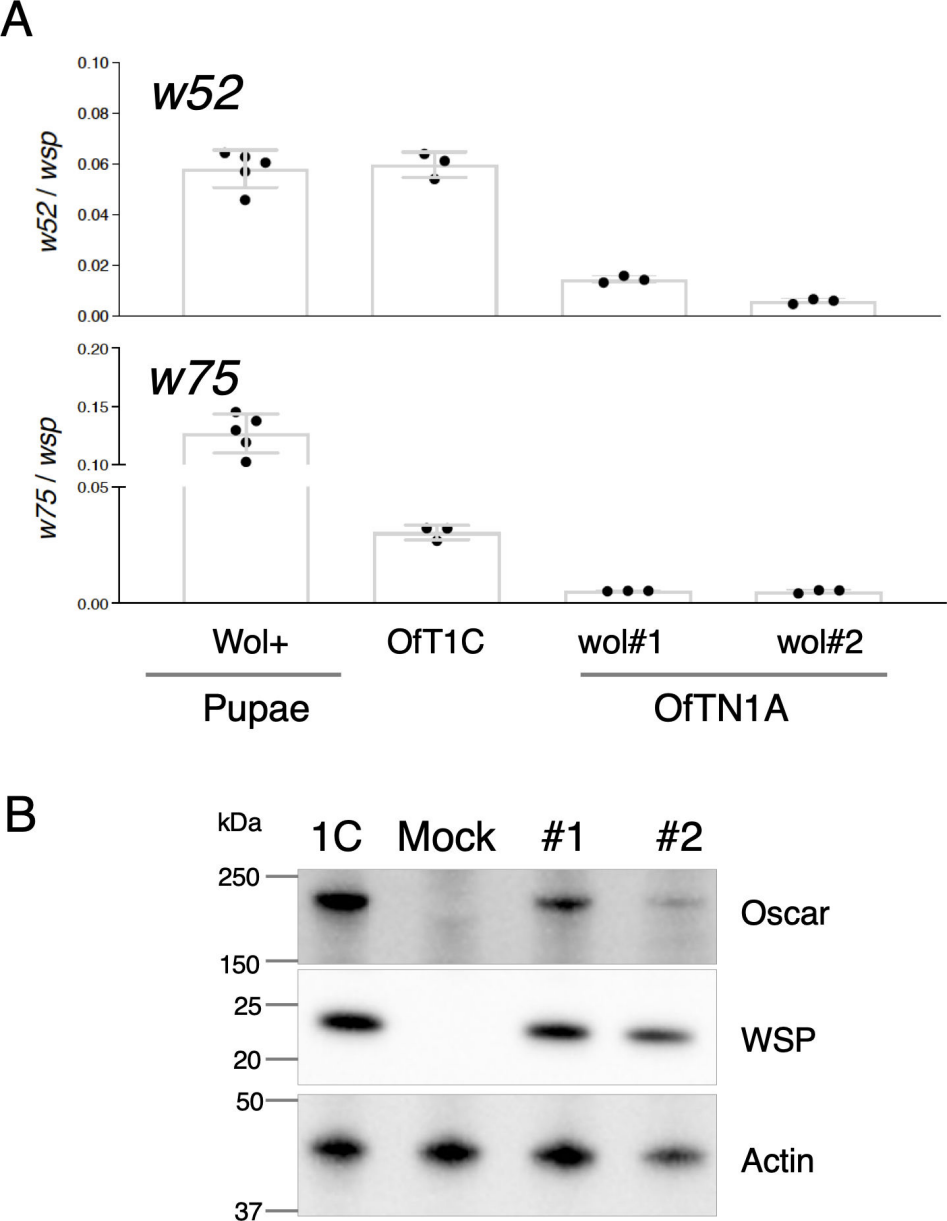
Expression of *w52* and *w75* in *O. furnacalis* pupal abdomens and cultured cells. (**A**) Expression of *w52* and *w75*. The mRNA levels of *w52* and *w75* in *O. furnacalis* pupal abdomens (*n* = 5) and cultured cells (triplicates) were examined by RT-qPCR. The mRNA level was normalized to that of *wsp*. Similar results were obtained in two independent experiments. Data shown are means ± SD. (**B**) Expression of WSP and Oscar proteins in *O. furnacalis* cultured cells. Western blotting of WSP and Oscar proteins was performed using cell lysates, with Actin as a loading control. Similar results were obtained in two independent experiments.

To assess whether *w52* and *w75* are actively transcribed during *w*Fur infection, we performed RT-qPCR using total RNA extracted from the pupal abdomens of *w*Fur-infected *O. furnacalis*, as well as from embryo-derived cultured cell lines OfT1C, OfTN1Awol#1, and OfTN1Awol#2. Transcripts corresponding to both genes were consistently detected across all *w*Fur-infected samples (Fig. 9A), confirming their expression and possible functional roles in infected cells. Quantitative analysis showed that the expression levels of *w52* and *w75* (normalized to *Wolbachia* house-keeping gene *wsp*) were higher in pupal tissues and OfT1C compared with those in artificially inoculated cell lines OfTN1Awol#1 and OfTN1Awol#2. Western blotting revealed that the abundance of Oscar, a *w*Fur-encoded male-killing effector (5), was substantially lower in the artificially inoculated cell lines compared with OfT1C cells (Fig. 9B). These findings strongly suggest that the expression levels of *Wolbachia* genes and proteins were lower in artificially inoculated cells (OfTN1Awol#1 and OfTN1Awol#2) than in a naturally infected (OfT1C) cell line.

### Effects of *w52* and *w75* expression on the proliferation of lepidopteran cultured cells

**Fig 10:**
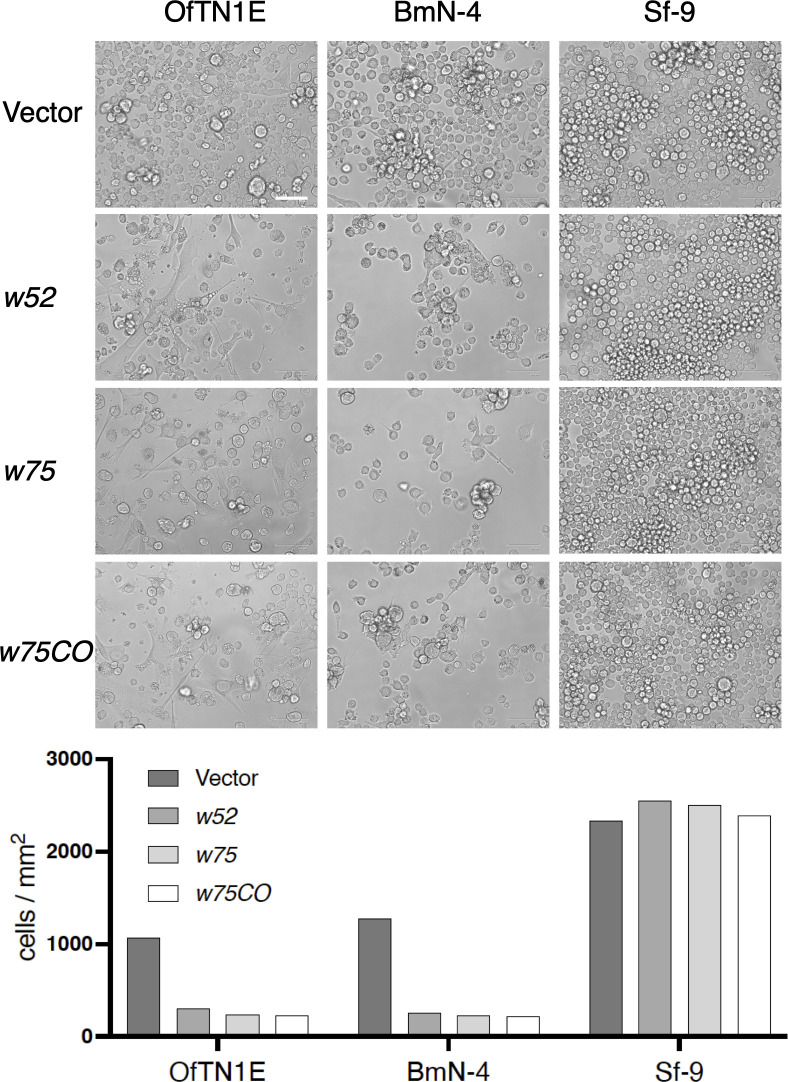
Effects of *w52* and *w75* expression on the growth of *O. furnacalis*, *B. mori*, and *S. frugiperda* cultured cells. Phenotypes of OfTN1E, BmN-4, and Sf-9 cells stably transfected with *w52*-, *w75*-, *w75CO*-expressing plasmids and empty vector were photographed. Bar, 100 µm. The average cell numbers counted from two images are shown below.

To assess whether the inhibitory effect of *w75* on cell proliferation depends on codon usage, we transfected OfTN1E cells with a codon-optimized version of *w75* (designated *w75CO*), tailored to the codon preference of *B. mori*. Transfection with *w75CO* similarly suppressed cell proliferation (Fig. 10), indicating that the codon bias of *w*Fur is not a key factor in the observed inhibition of cell proliferation. Moreover, consistent with the results in *O. furnacalis* cells, overexpression of *w52* and *w75* also led to marked suppression in the proliferation of *Bombyx mori*-derived BmN-4 cells (Fig. 10). In contrast, transfecting these genes into *Spodoptera frugiperda* Sf-9 cells had no impact on cell proliferation, which remained comparable to that of cells transfected with the empty vector (Fig. 10). These results suggest that the inhibitory effects of *w52* and *w75* are not broadly conserved across lepidopteran species or cell lines, implying species- and/or cell type-specific responses to these *w*Fur-encoded proteins.

### Cellular localization of W75 and W52 proteins

**Fig 11:**
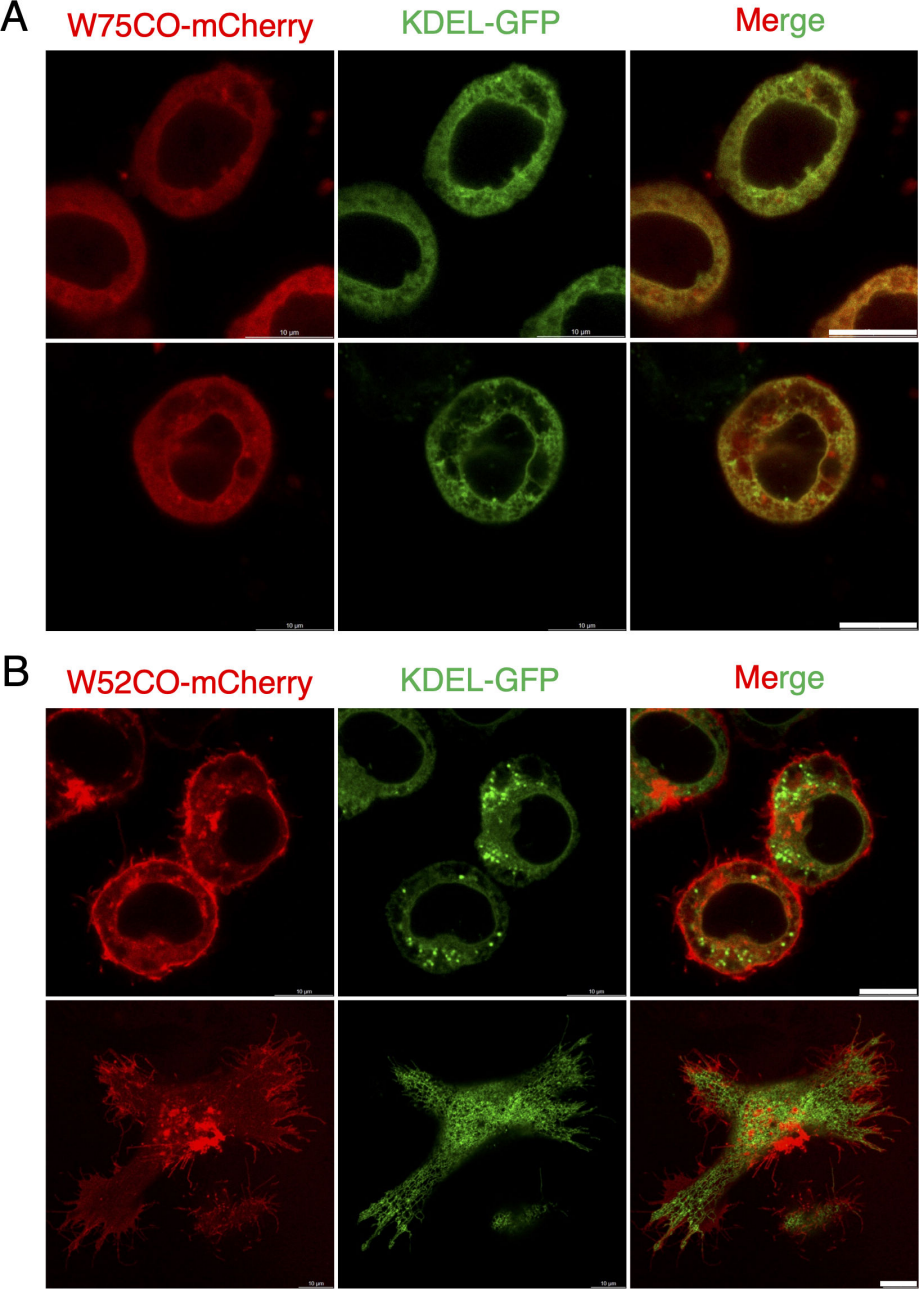
Intracellular localization of W52 and W75 proteins. OfTN1E cells were co-transfected with the *KDEL-EGFP* and *w75CO-mCherry* (**A**) or *w52CO-mCherry* (**B**) constructs, and the localization of EGFP- and mCherry-fused proteins was examined. Bar, 10 µm.

To investigate the subcellular localization of the W75 and W52 proteins, we transfected *O. furnacalis* OfTN1E cells with a construct encoding a codon-optimized version of *w75* or *w52* fused to *mCherry* (*w75CO-mCherry* or *w52CO-mCherry*). Confocal microscopy showed that W75CO-mCherry was predominantly localized in the cytoplasm, with marked accumulation in the perinuclear region (Fig. 11A). To further characterize its localization, co-transfection was performed with KDEL-EGFP, a fluorescent marker carrying the endoplasmic reticulum (ER) retention signal (KDEL). The EGFP and mCherry signals overlapped to some extent, indicating that the W75 protein is partially localized to the ER in transfected cells (Fig. 11A). In contrast, W52CO-mCherry was predominantly localized in the plasma membrane, and its localization did not overlap with that of KDEL-EGFP (Fig. 11B).

## DISCUSSION

In this study, we demonstrated that inoculation with *w*Fur markedly reduces the proliferation of *O. furnacalis* cultured cells, accompanied by cytotoxic effects, aberrant morphology, and cell cycle arrest. Moreover, we successfully identified two candidate genes potentially involved in this phenotype. One of these, *w52*, is a homolog of the previously characterized gene *TomO*, originally identified in *w*Mel (15). *TomO* was first discovered through transfection of *w*Mel genomic fragments into *D. melanogaster* S2 cells, where it suppressed cell proliferation. Subsequent investigations revealed that TomO binds host *nanos* mRNA, enhancing Nanos protein production and thereby promoting the expansion of germline stem cells in *D. melanogaster* (15). Phylogenetic analysis indicated that *w52* is exclusively conserved within supergroup B, suggesting that *TomO* homologs possess supergroup B *Wolbachia*-specific functions. The *w*Pip TomO has also been implicated in germline stem cell proliferation in transgenic *D. melanogaster* (19). Combined with our observation of high-level *w52* expression in *O. furnacalis* pupal abdomens (Fig. 9A), which contain numerous developing ovarioles, and in *O. furnacalis* embryo-derived OfT1C cells, W52 may also play a role in modulating germline stem cell dynamics in lepidopteran hosts by enhancing Nanos expression. In addition, given the RNA-binding activity of TomO (15), its homolog W52 likely possesses similar activity, and its expression in *Wolbachia*-inoculated or *w52*-transfected cells presumably disturbs or alters intracellular RNA dynamics and functions, ultimately leading to inhibition of cell proliferation.

The second gene, *w75*, represents a previously uncharacterized *Wolbachia* gene that appears to be conserved across diverse *Wolbachia* lineages (Fig. 7B; Fig. S5). Similar to *w52*, *w75* encodes a transmembrane protein, and its overexpression in *O. furnacalis* cultured cells results in partial localization to the ER (Fig. 11A). In contrast to *w52*, transient overexpression of *w75* significantly increased caspase activity in *O. furnacalis* cultured cells (Fig. 8B), which may contribute to the inhibition of cell proliferation during *w*Fur infection. Expression of *w75* or *w52* alone did not affect LDH activity; however, co-transfection of *w52* and *w75* significantly increased LDH activity, suggesting that these two genes exert additive or synergistic effects in inducing cell membrane damage, thereby promoting LDH release. Although both W52 and W75 expression exhibited cell proliferation-inhibitory activity *in vitro*, their precise biological roles during *w*Fur endosymbiosis in *O. furnacalis* remain unresolved and warrant further investigation.

There have been relatively few reports on the transinfection of *Wolbachia* into lepidopteran cultured cells. Recently, experiments were reported in which various *Wolbachia* strains, including *w*Sca from *O. scapulalis*, a close relative of *O. furnacalis*, were introduced into male *O. scapulalis*-derived cultured cells to investigate the *Wolbachia*’s ability to suppress masculinization by analyzing the splicing patterns of sex-determining genes (20, 21). However, these reports did not describe the condition of the inoculated cells, such as whether cell proliferation was affected. Similarly, a study transplanting *Wolbachia* from *Eurema hecabe* into *B. mori* cultured cells has been documented, but again, no effects on cell proliferation or cytotoxicity were reported (22). In our study, we transplanted the male-killing *Wolbachia* strain *w*Fur, originally isolated from *O. furnacalis*, into cultured cells derived from the same host species and observed a marked suppression of cell proliferation (Fig. 1 and 2). This phenotype was consistently observed in two distinct cell lines, suggesting that inhibition of cell proliferation may represent a characteristic feature of *w*Fur, at least in *O. furnacalis* embryonic cultured cells. Moreover, given the high genomic similarity between *w*Sca and *w*Fur, including the presence of both *w52* and *w75*, it is plausible that *w*Sca may exhibit a similar infection phenotype. We intend to test this hypothesis through future transplantation experiments using *w*Sca-containing inocula.

We conducted a functional screen of *Wolbachia* genes without applying codon optimization for the host. Fortuitously, two genes exhibiting cell proliferation-inhibitory activity were identified, one of which corresponds to a previously reported gene known to suppress proliferation in *D. melanogaster* S2 cells (15). Moreover, the codon-optimized *w75* for *B. mori* showed inhibitory effects on cell proliferation comparable to those of the native *w75*, thereby validating its functional activity. Typically, when expressing *Wolbachia* genes in insect hosts or cultured cells, synthetic genes optimized for host codon usage are employed (3–5, 15), as native sequences often yield insufficient expression. To improve the efficiency of large-scale screens, such as the present study, it will be important to develop a system that enables robust expression of *Wolbachia* gene products directly from library clones without codon optimization. Establishing such a platform remains a critical challenge for future research.

## MATERIALS AND METHODS

### Insects and cell lines

*w*Fur-infected and *Wolbachia*-free *O. furnacalis* larvae were reared on an artificial diet (Insecta LFS, Nosan Corp., Japan) at 23°C–25°C under a photoperiod of 16L:8D. *O. furnacalis* cell lines OfTN1A and OfTN1E were established from *Wolbachia*-free *O. furnacalis* embryos as previously described, with minor modifications (5). OfTN1A, OfTN1E, and *w*Fur-infected OfT1C cells were maintained at 26°C in Express Five SFM (Gibco, USA) supplemented with 18 mM L-glutamine and 10% fetal bovine serum (FBS) (Gibco). OfT1C/tet cells were reported previously and were cultured in the same medium supplemented with 3 µg/mL tetracycline (5).

### *Wolbachia* transinfection

The scheme of *Wolbachia* transinfection is summarized in Fig. 1A. Forty milliliters of OfT1C or OfT1C/tet cells (~2.0 × 10^7^ cells) was centrifuged at 2,000 rpm, and the resulting cell pellets were resuspended vigorously in 10 mL of FBS-free Express Five SFM using a 5-mL pipette. The suspension was passed twice through a 27-gauge syringe and subsequently filtered through a 5-µm filter, followed by a 1.2-µm filter. Then, 1 mL of the filtered solution was inoculated onto OfTN1A or OfTN1E cells (4.0 × 10^5^ cells in a T25 flask). OfTN1A treated with 0.2 mL of OfT1C-derived solution and 0.8 mL of OfT1C/tet-derived solution was designated OfTN1Awol#1, whereas that treated with 1 mL of OfT1C-derived solution was designated OfTN1Awol#2. On the following day, the medium was replaced with fresh Express Five SFM/FBS, with or without tetracycline.

### Reverse transcription-PCR (RT-PCR) and quantitative PCR (qPCR)

Total RNA was prepared from *O. furnacalis* pupal abdomens and cultured cells using TRI REAGENT (Molecular Research Center Inc., USA). First-strand cDNA was synthesized from 500 ng of total RNA using avian myeloblastosis virus reverse transcriptase with both oligo-dT and random primers (TaKaRa, Japan).

Quantitative RT-PCR (RT-qPCR) of *w52* and *w75* was performed using SYBR Green qPCR Master Mix (Thermo Fisher Scientific, USA) with the following primers: w52-qF3: 5′-GATCTTGCACAAATAATAGC-3′ w52-qR3: 5′-TTCCACTGTGTCAATTACAG-3′ w75-qF: 5′-TTTGAAGAGGTATTTAGTGC-3′ w75-qR: 5′-CAAGCTTAACTTCCTGATAG-3′.

Primers for *Ofrps3* and *wsp* were reported previously (5). mRNA levels were normalized to *Ofrps3*, and expression values were calculated using the 2^−ΔΔCt^ method.

### Estimation of *Wolbachia* density

Genomic DNA was extracted from *O. furnacalis* cultured cells using the DNeasy Blood & Tissue Kit (QIAGEN, Germany). *Wolbachia* density was estimated by qPCR for *wsp*. qPCR was carried out using SYBR Green qPCR Master Mix (Thermo Fisher Scientific). Relative values were calculated using the 2^−ΔΔCt^ method, with normalization to *O. furnacalis EF-1α,* as reported previously (5).

### Estimation of chromosome numbers by qPCR

Genomic DNA was extracted from 3.5 × 10^5^ *O. furnacalis* cultured cells and subjected to genomic qPCR. qPCR of three autosomal genes, *O. furnacalis EF-1α* (5), *SOX-5*, and *ATPase13A3*, was performed using SYBR Green qPCR Master Mix (Thermo Fisher Scientific). The primers for amplification of *SOX-5* and *ATPase13A3* are as follows: OfSOX5-F, 5′-ACCAGGACCTCCATCAGCGA-3′; OfSOX5-R, 5′-GTCCCAACACCTTGGCCTCA-3′; OfATPase13A3-F, 5′-GGCGTAGTTCTCCCAACATTCGT-3′; OfATPase13A3-R, 5′-GGCATGACGGAGGCTTATACCG-3′.

### Caspase assay

*O. furnacalis* cultured cells (1.3 × 10^4^ cells/well in a 96-well black plate) were subjected to measure caspase activity using Caspase-Glo3/7 assay kit (Promega, USA) according to the manufacturer’s protocol.

### LDH assay

*O. furnacalis* cultured cells (1.3 × 10^4^ cells/well in a 96-well plate) were subjected to measure LDH activity using Cytotoxicity LDH Assay Kit-WST (Dojindo Molecular Technologies, Japan) according to the manufacturer’s protocol.

### FACS analysis

Cell cycle analysis was performed using propidium iodide (PI) staining. *O. furnacalis* cultured cells (5 × 10^5^ cells) were seeded in a T25 flask and cultured for 2 days. Next, cells were collected and fixed in cold 70% ethanol for 4–24 h at 4°C. Cells were then washed once with phosphate-buffered saline (PBS) and incubated with 500 μL of PI staining solution (10 μg/mL PI, 100 μg/mL RNase in PBS) for 30 min in the dark. Cell cycle analysis was carried out using a CytoFLEX (BECKMAN COULTER, USA) and Kaluza Analysis 2.1 (BECKMAN COULTER).

### Plasmid construction, transfection, and zeocin treatment

An expression library containing ~300 *w*Fur-derived clones, which contained *Wolbachia*-specific and ANK-containing genes, was constructed using pIZ/V5-His-g3 (23) or pIZ/V5-His (Invitrogen). The codon-optimized *w52* (*w52CO*) and *w75* (*w75CO*) fragments were synthesized by Eurofins Genomics (Germany) and cloned into pIZ/V5-His-g3. OfTN1E, BmN-4, or Sf-9 cells were transfected with 1 µg of each plasmid DNA using X-tremeGENE HP (Roche Applied Science) or FuGENE HD (Promega, USA) and treated with zeocin (500 µg/mL for OfTN1E and BmN-4 cells, 1,000 µg/mL for Sf-9 cells) at 3 days post-transfection. At 11–13 days post-transfection, cell growth and morphology were assessed using a FLoid Cell Imaging Station (Life Technologies, USA).

### Cellular localization of W52 and W75 proteins

*w52CO* and *w75CO* were cloned into pIZ/V5-His-g3-mCherry (24) and transfected into BmN-4 or OfTN1E cells (4 × 10^5^ cells per 35-mm diameter dish), with or without pIZ/V5-His-g3-KDEL-EGFP, which expresses a modified EGFP derivative with ER retention signal (KDEL) at the C-terminus and Bombyx mori nucleopolyhedrovirus chitinase signal peptide at the N-terminus. The localization of EGFP- and mCherry-fused proteins was examined using an EVOS M5000 cell imaging system (Thermo Fisher Scientific) and a Leica STELLARIS5 confocal microscope (Leica, Germany) with an HC PLAPO CS2 20× DRY objective lens (N.A. = 0.75), HC PL APO CS2 63×OIL objective lens (N.A. = 1.40), and HyD detectors.

### Phylogenetic analysis

Complete genome sequences and protein sequences of 311 *Wolbachia* lineages were retrieved from NCBI RefSeq. Single-copy orthologs were identified with OrthoFinder v3.1.0 (25), and their amino acid sequences were aligned using MAFFT v7.525 (26), trimmed with trimAl v1.5 (27), and concatenated for phylogenetic analysis, yielding an alignment of 55,036 amino acids from 203 single-copy genes. Phylogenetic inference was performed with IQ-TREE v2.3.6 (28) without partitioning, and the best-fit substitution model was estimated by ModelFinder implemented in IQ-TREE. To assess conservation of *w52* and *w*75, blastn and blastp searches were performed using their nucleotide and amino acid sequences, respectively, as queries against the genome and protein sequences of 311 lineages, with an E-value cutoff of 1e-02. For each lineage, the top hit (lowest E-value) was selected. Phylogenetic trees and blast results were visualized with iTOL (29).

### Western blotting

Collected cells were lysed on ice for 15 min in chilled TNE-N buffer (20 mM Tris pH 8.0, 1 mM EDTA, 150 mM NaCl, 1% NP-40) supplemented with cOmplete EDTA-free (Roche, Switzerland). Lysates were centrifuged at 20,000 × *g* for 15 min at 4°C, and aliquots of the supernatant were mixed with 2× SDS sample buffer (4% SDS, 20% glycerol (liquid), 125 mM Tris-HCl (pH 6.8), 0.04% BPB, 0.2 M DTT). Samples were boiled for 5 min, and proteins were separated on 4%–12% Bis-Tris gels (NuPAGE, Invitrogen, USA) in MOPS buffer using an XCell SureLock mini-cell (Invitrogen, USA). The proteins were transferred to PVDF membranes using an XCell II blot module (Invitrogen, USA) according to the manufacturer’s protocol. The membranes were blocked with 4% Block Ace (DS Pharma Biomedical, Japan) and incubated with primary antibodies in antibody dilution buffer: anti-WSP-2 antibody (Eurofins Genomics, Japan, raised against a synthetic peptide [a.a. 147–165 of WSP] 1:2,000 dilution), anti-Oscar antibody (Eurofins Genomics, Japan, raised against a synthetic peptide [a.a. 1,512–1,525 of Oscar] 1:2,000 dilution) or anti-actin antibody (sc-1616-R: 1:2,000 dilution, Santa Cruz, USA) in antibody dilution buffer “Kiwami Setsuyaku-kun” (DRC, Japan). After incubation with the primary antibody, the membranes were washed four times with TBS-T buffer and incubated with the secondary antibody: HRP-conjugated anti-Rabbit IgG (111-035-144, Jackson ImmunoResearch Laboratories Inc., USA; 1:10,000 dilution). After incubation with the secondary antibody, the membranes were washed five times with TBS-T buffer and developed using SuperSignal West Pico PLUS Chemiluminescent Substrate (Thermo Fisher Scientific, USA). Protein bands were detected using a ChemiDoc XRS Plus imaging system (Bio-Rad, USA).

## SUPPLEMENTAL MATERIAL

Supplemental figures mbio.00074-26-s0001.pdf Fig. S1 to S5.

ASM does not own the copyrights to Supplemental Material that may be linked to, or accessed through, an article. The authors have granted ASM a non-exclusive, world-wide license to publish the Supplemental Material files. Please contact the corresponding author directly for reuse.
