# Supplementary material for: Identification of two Wolbachia genes with cell proliferation-inhibitory activity in Ostrinia cultured cells
Source: mBio. 2026 Mar 30;17(5):e00074-26. doi: 10.1128/mbio.00074-26 (PMC13170316; doi:10.1128/mbio.00074-26)
Supplement: Supplemental figures — Fig. S1 to S5. [file mbio.00074-26-s0001.pdf]

Fig.S1

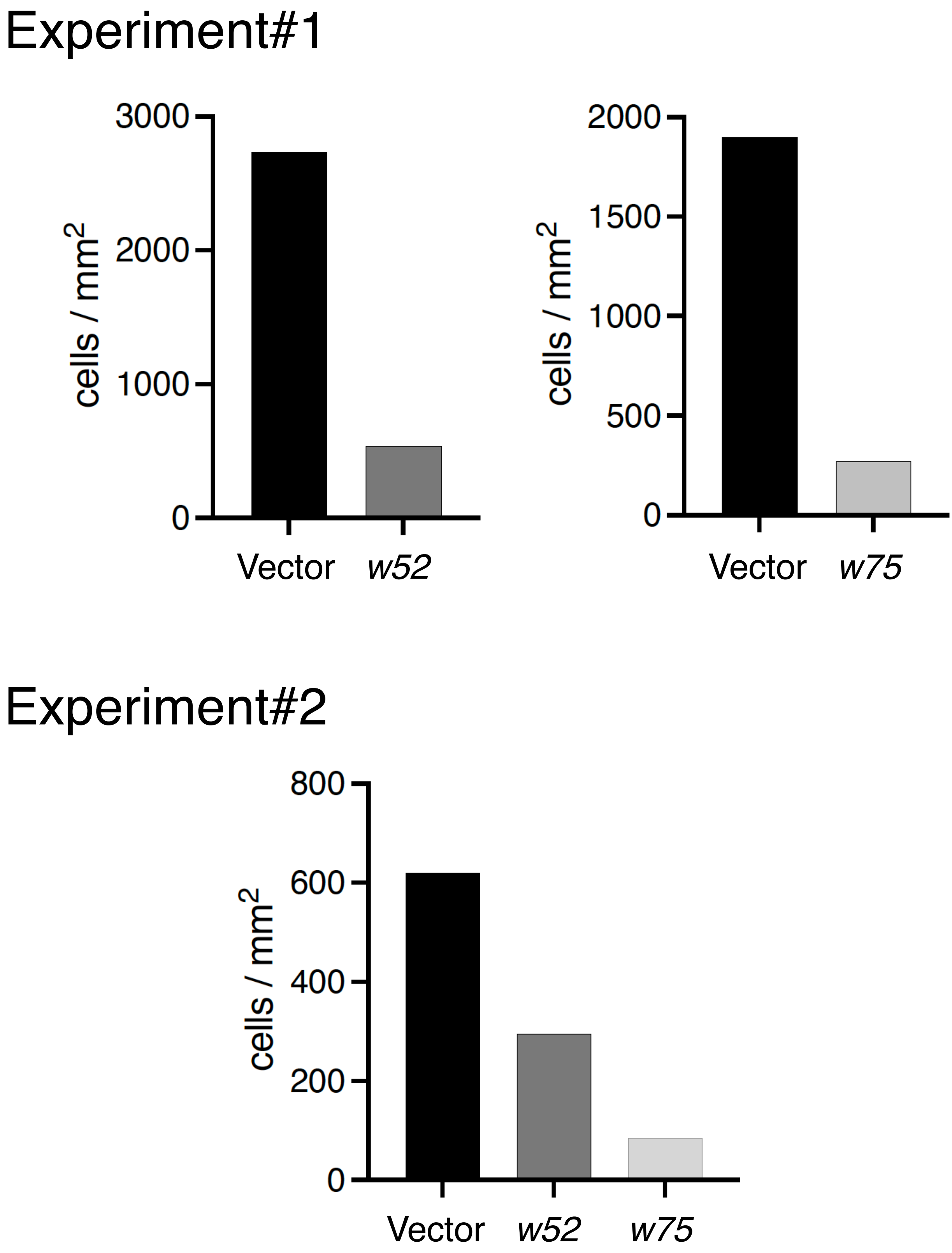

Fig. S1. Stable transfection of OfTN1E cells with *w52*- or *w75*-expressing plasmid, related to Fig. 5. OfTN1E cells were transfected with empty vector, or plasmids containing *w52* or *w75*, selected with zeocin, and photographed at 11–13 days after transfection. The average cell numbers counted from 2–4 images at each experiment are shown.

Fig.S2

```
w52-full       ------------------------------------------------------------
w52-cloned     ------------------------------------------------------------
TomO-wPip      MGWNLLGGLMPPAVDTAEERERRHSRDSGAEEEFEVLEQEEIKEHRHSGDSGNCSEVEEG

w52-full       ------------------------------------------------------------
w52-cloned     ------------------------------------------------------------
TomO-wPip      QNTNRAIELEFQLTQSLLSQVKAGENNRNANSGKQAQLDLERMDFVINGQTISKAYVHEL

w52-full       ------------------------------------------------------------
w52-cloned     ------------------------------------------------------------
TomO-wPip      YCQHNHLISSENANYRLFAKQVFTEMFKYAKAETPNDNILEELITNCNQAGYDGSLLMQL

w52-full       ---------------------------------------MVKQLGKEICRVDAILEFTLK
w52-cloned     ------------------------------------------------------------
TomO-wPip      SPIFSKHKLSLPHANDRKISIVYNSQGSLNIQYCPSMPVRELDTNKEICRVDAILEFTLK

w52-full       FRDSKVEYENGKVIFAIPEQLENYKADGKSLLDEINRHFEDEDNRITETLEKNMNEGPKS
w52-cloned     -----------------------------------------------------MNEGPKS
TomO-wPip      FRDSKVEYENGKVTLAIPEQLENYKVDGKSLLDEINRHFEDEDNRITETLEKNMNEGPKS
                                                                    *******

w52-full       FAAEDLSITESSSDSSEVANKVVTDPGILGNVANVLVGVAEVTAVVATELFWPQDQINIE
w52-cloned     FAAEDLSITESSSDSSEVANKVVTDPGILGNVANVLVGVAEVTAVVATELFWPQDQINIE
TomO-wPip      FAAEDLSITESSSDSSEVANKVVTDPGILGNVANVLVGVAEVTAVVATELFWPQDQVNIE
               ********************************************************:***

w52-full       PRHNEEEFTIIPENLTFDGLIDVINKAKNYGDYADLDKTINNIEESDRFITDLNCGNLKD
w52-cloned     PRHNEEEFTIIPENLTFDGLIDVINKAKNYGDYADLDKTINNIEESDRFITDLNCGNLKD
TomO-wPip      PRHNEEEFTITPKNLTFDGLIDVINKAKNYGDYADLDKTINNIEESDRFITDLNCGNLKD
               ********** *:***********************************************

w52-full       FCSQQLNLTDHMHNVLLNNSYDETNGYYNYSVKELSLLVIAADDTLVERYAETHKSILFT
w52-cloned     FCSQQLNLTDHMHNVLLNNSYDETNGYYNYSVKELSLLVIAADDTLVERYAETHKSILFT
TomO-wPip      FCSQQLNLTDHMHNVLLNNSYDETNGYYNYSVKELSLLAIAADDTLVERYAETHKSILFT
               **************************************.*********************

w52-full       QDEGLDRLLIGVGGQRYTKLLLEDLEKNRSTILPWHSICEKLRRALSIRNQECVKAVLDK
w52-cloned     QDEGLDRLLIGVGGQRYTKLLLEDLEKNRSTILPWHSICEKLRRALSIRNQECVKAVLDK
TomO-wPip      QDEGLDRLLIGVGGQRYTKLLLEDLEKNRSTILPWHSICEKLRRALSIRNQECVKAVLDK
               ************************************************************

w52-full       FKDTEARSRVSDMSEKFLHYAIQQDFIDVAKTIINHKIADINSIDNSGHAPLHWAVARNN
w52-cloned     FKDTEARSRVSDMSEKFLHYAIQQDFIDVAKTIINHKIADINSIDNSGHAPLHWAVARNN
TomO-wPip      FKDTEARSRVSDMSEKFLHYAIQQDFIDVAKTIINHKIADINSIDNSGHAPLHWAVARNN
               ************************************************************

w52-full       LELIGLLIKNGANVDVQDERHGRTALHWAAYHDKFEIVKLLVNKGADCNIKDRDGKTALD
w52-cloned     LELIGLLIKNGANVDVQDERHGRTALHWAAYHDKFEIVKLLVNKGADCNIKDRDGKTALD
TomO-wPip      LELMGLLIGNGAEIDIQDERHGRTALHWAAYHDKFEIVKLLVNKGADWNIQDRDGKTALD
               ***:**** ***::*:******************************* **:*********

w52-full       LVGTKSLYLVREENREKSSSESKITKFLEGLDDKTPLHLNIKKDPESKNIDNQQPQRNIV
w52-cloned     LVGTKSLYLVREENREKSSSESKITKFLEGLDDKTPLHLNIKKDPESKNIDNQQPQRNIV
TomO-wPip      LVGTKSLYLVREENREKSSSESKITKFLEGLDDKTPLHLNIKKDPESKNIDNQQPQRNIV
               ************************************************************

w52-full       DKVENIMNNEEASIEEAIGNNCEEGKMPGNEKIIGEKLSDAVDRINRDTMIDLLKKLDDS
w52-cloned     DKVENIMNNEEASIEEAIGNNCEEGKMPGNEKIIGEKLSDAVDRINRDTMIDLLKKLDDS
TomO-wPip      DKVENIMNNEEASIEEAIGNNCEEGKMPGNEKIIGEKLSNAVDRINLTRLIHLLKKLEVS
               ***************************************:******   :*.*****: *

w52-full       VYKSTLQKLLSQANDKMTSLGEGKQKEDLAQIIALLKNELEHIDASS-VGNDVEANASGQ
w52-cloned     VYKSTLQKLLSQANDKMTSLGEGKQKEDLAQIIALLKNELEHIDASS-VGNDVEANASGQ
TomO-wPip      TYEPTVQKLLDQANDKITNLEEGEQKEALEKIILFLENELKHTAATLPVDNNVEAAASSQ
               .*:.*:****.*****:*.* **:*** * :** :*:***:*  *:  *.*:*** **.*

w52-full       QIPVEKVKERESDSPSLSSEDDDFSKLEGNKSDCSAGTSSDSNSNSPFEKISEEGHETRI
w52-cloned     QIPVEKVKERESDSPSLSSEDDDFSKLEGNKSDCSAGTSSDSNSNSPFEKISEEGHETRI
TomO-wPip      EAPLVESKE---SSPSLSSEEDDFSKLEGNKSDCSAGTSSDSNSNSPFEKISEEGHETRI
               : *: : **   .*******:***************************************

w52-full       SVIDTVEAEATANGTVSSNSVLCTLRVELTSDDEDELLSIEIEDVNNNINVNQPTKHVEQ
w52-cloned     SVIDTVEAEATANGTVSSNSVLCTLRVELTSDDEDELLSIEIEDVNNNINVNQPTKHVEQ
TomO-wPip      SVIDTVEAEATANGTVSSNSVPCTLRVELTSDDEDEFLSIKIEDVNNDINVNQPTEHVEQ
               ********************* **************:***:******:*******:****

w52-full       VANGNGQPKTELPRINEQSSKSGLKKNPQNTKKYVVAASALAIAGIVSGVAVAVYLEMLA
w52-cloned     VANGNGQPKTELPRINEQSSKSGLKKNPQNTKKYVVAASALAIAGIVSGVAVAVYLEMLA
TomO-wPip      VANGNGQPKTELPRINEQSSKSGLKKNPQNTKKYVVAASALAIAGIVSGVAVAVYLEMLA
               ************************************************************

w52-full       VGIAVAACCLIAATVTYCCRPKSLIEDNQVKKVMQTEECQSK
w52-cloned     VGIAVAACCLIAATVTYCCRPKSLIEDNQVKKVMQTEECQSK
TomO-wPip      VGIAVAACCLIAATVTYCCRPKSLIENGEPAEIVFKERSVY-
               **************************:.:  ::: .*..
```

Fig. S2. Amino acid sequence alignment of W52 and TomO$_{wPip}$. The cloned *w52* lacked a 222 base pair-long 5' region potentially encoding a 74 aa-long amino acids (underlined in red) of the N-terminus of the W52 protein.

Fig.S3

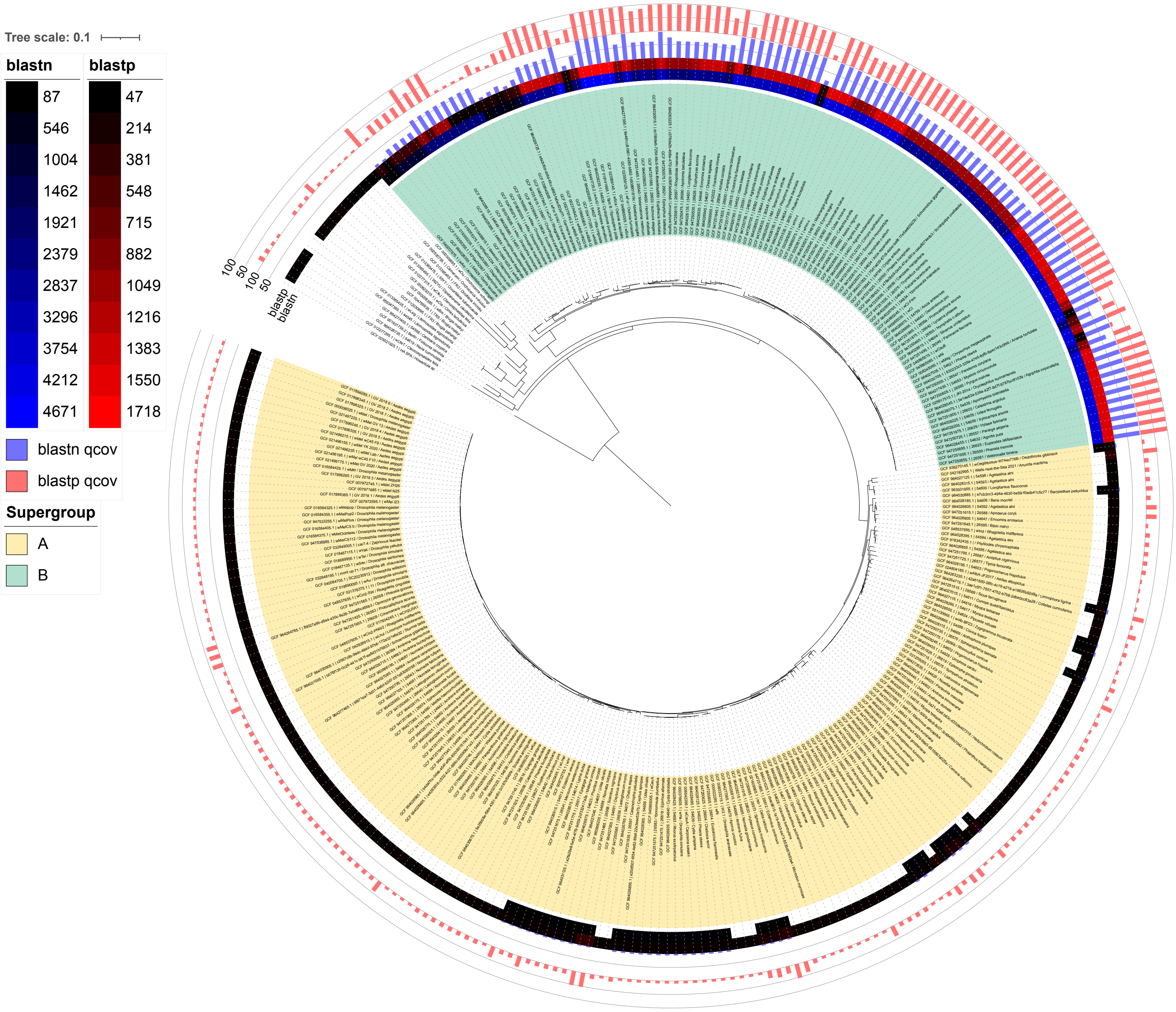

Fig. S3. Phylogenetic distribution of *w52* homologs across *Wolbachia* genomes. Complete phylogenetic tree corresponding to Fig. 7A, including all lineages, is shown.

Fig.S4

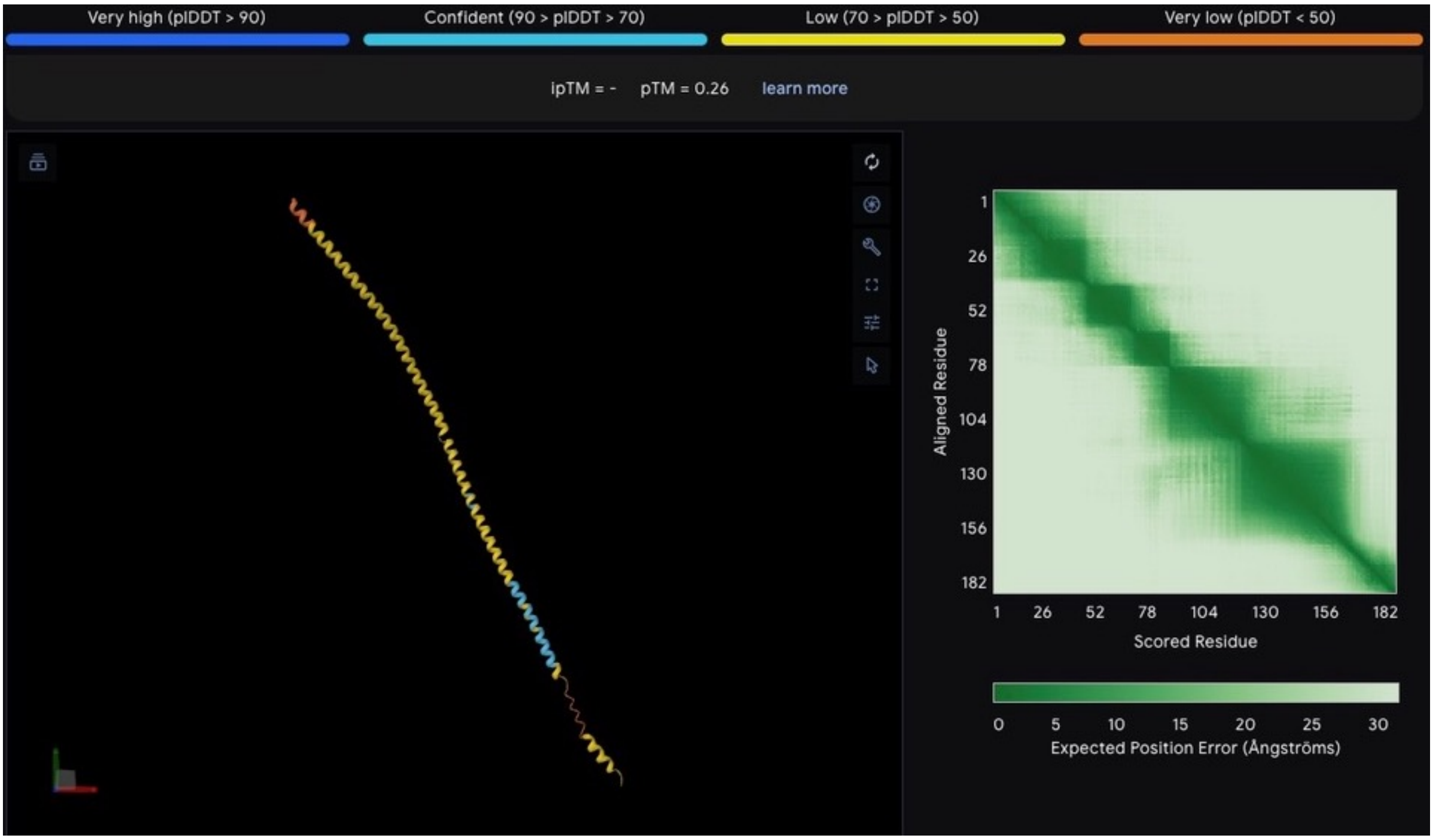

Fig. S4. Structure prediction of the W75 protein. The structure of the W75 protein was predicted by AlphaFold3. A heatmap of predicted aligned errors (PAEs) is also shown. AlphaFold3 has high confidence when visualized in green.

Fig.S5

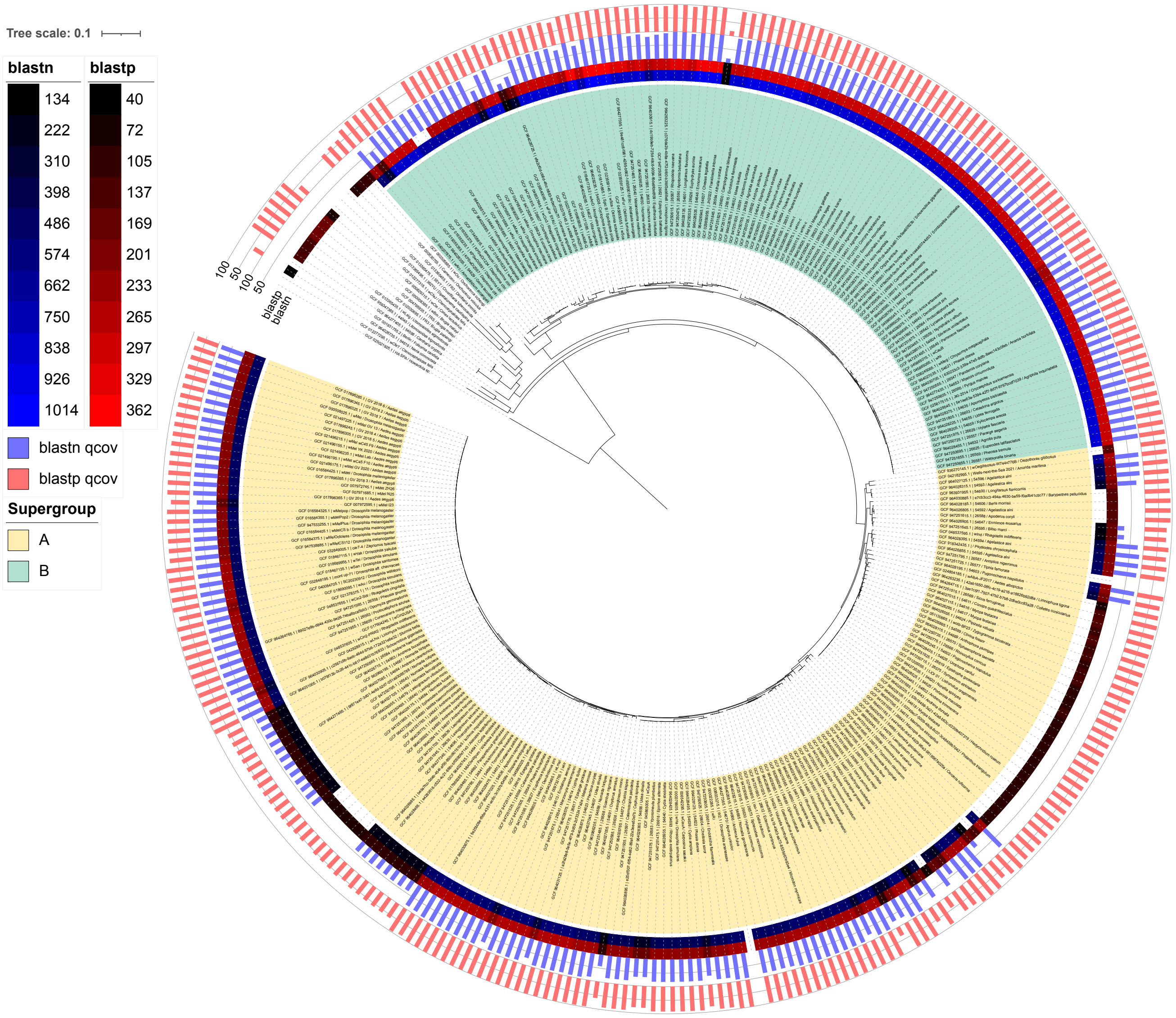

Fig. S5. Phylogenetic distribution of *w75* homologs across *Wolbachia* genomes. Complete phylogenetic tree corresponding to Fig. 7B, including all lineages, is shown.
